# Effect of vitamin D3 on self-perceived fatigue: A double-blind randomized placebo-controlled trial: Erratum

**DOI:** 10.1097/MD.0000000000006038

**Published:** 2017-01-20

In the article “Effect of vitamin D3 on self-perceived fatigue: A double-blind randomized placebo-controlled trial”,^[1]^ which appeared in Volume 95, Issue 52 of *Medicine*, Drs. Suter, Battegay, and Schmid were incorrectly reported as having PhDs. In fact, all three authors are MDs.
